# Achilles Tendon Reconstruction Using a Hamstring Tendon Autograft for Chronic Rupture of the Achilles Tendon in Patients Over 70 Years of Age: A Retrospective Case Series

**DOI:** 10.7759/cureus.42788

**Published:** 2023-08-01

**Authors:** Yasunari Ikuta, Tomoyuki Nakasa, Shingo Kawabata, Nobuo Adachi

**Affiliations:** 1 Department of Orthopedic Surgery, Graduate School of Biomedical and Health Sciences, Hiroshima University, Hiroshima, JPN

**Keywords:** geriatric population, older patients, autografting, hamstring tendon, chronic achilles tendon rupture

## Abstract

Reconstruction techniques using autologous hamstring tendons were generally applied for chronic Achilles tendon rupture with a large defect size. Previous studies have reported good clinical results of this technique for young or middle-aged patients, however, the clinical outcomes in older patients have been unclear. This retrospective case series reviewed four male patients aged >70 years (mean age, 78.5 years) who underwent Achilles tendon reconstruction using the hamstring tendon autograft for chronic rupture of the Achilles tendon with a large tendon defect. The proximal-distal length between the healthy tendon stumps was measured using sagittal T2-weighted magnetic resonance imaging (MRI). The American Orthopedic Foot and Ankle Society (AOFAS) ankle-hindfoot score and postoperative complications were evaluated. The duration from the traumatic event or appearance of symptoms to surgery was 3.8 (range, 2-6) months. The mean measured gap between the healthy tendon stumps was 67.5 mm on MRI. The AOFAS ankle-hindfoot score improved from 67.3 to 99.5 at the mean follow-up period of 40.3 (range, 23-75) months. No donor site morbidity was observed in all patients. Re-rupture was detected at the five-month follow-up in one patient who had removed a hinged ankle-foot orthosis with adjustable heel wedges without permission. Achilles tendon reconstruction using a hamstring tendon is a viable option for treating selected patients with chronic rupture of the Achilles tendon with a large tendon defect even in older patients. To improve clinical outcomes, a better understanding should be provided to family members as well as older patients regarding the postoperative rehabilitation program.

## Introduction

Achilles tendon rupture is a common tendon injury in the adult population, and its incidence has increased over the past decade [1]. Recent epidemiologic studies have demonstrated that the incidence of Achilles tendon rupture ranges from 21.5 to 31.2 per 100,000 person-years [1,2]. The mean age of patients with Achilles tendon rupture has also been increasing due to rising opportunities to participate in recreational sports, particularly in older adults [3]. Initial misdiagnosis is common in older patients [4].

Chronic Achilles tendon rupture is defined as a rupture with delayed diagnosis > 4 weeks from the initial injury [5]. In general, most patients pursue reconstruction to restore functional length, tension, and strength to the gastrocnemius-soleus complex to improve gait and function [6]. Several surgical techniques have been described for treating chronic Achilles tendon ruptures, such as primary repair, V-Y advancement, turndown flap, tendon transfer, tendon autograft, allograft, and synthetic materials [3,6,7]. These types of surgical management are based on a treatment algorithm that focused on the length of the tendon defect [6,8].

Reconstruction techniques using autologous hamstring tendons were generally employed for chronic Achilles rupture with a large defect size [7]. Achilles tendon reconstruction using a semitendinosus tendon autograft yielded good midterm results in young adulthood or middle-aged patients via open procedure [9], a less-invasive approach [10], and endoscopic techniques [11]. Autologous gracilis tendon grafts also provided good clinical and functional outcomes at a mean follow-up of 10.9 years [12]. Although previous studies have reported good clinical results of Achilles tendon reconstruction for chronic rupture using hamstring tendons, such studies focused on only young or middle-aged patients. The clinical outcomes of reconstruction using an autologous hamstring tendon in older patients have not been reported in the literature. Therefore, we focused on older patients who were treated with a hamstring tendon autograft for chronic Achilles tendon rupture with a large tendon defect. Here, we show four cases of reconstruction using a hamstring tendon autograft for chronic rupture of the Achilles tendon in patients aged ≥70 years.

## Case presentation

This study was approved by the ethics committee of our university. This retrospective analysis included four men aged ≥70 years (mean age, 78.5; range, 71-84) who underwent Achilles tendon reconstruction using the hamstring tendon for chronic rupture of the Achilles tendon. All patients were diagnosed with chronic Achilles tendon rupture at primary clinics and were referred to our hospital for surgical treatment. Chief complaints, injury mechanism, cause of chronicity, and the durations from the traumatic event or appearance of symptoms to surgery were reviewed.

Surgical treatment was indicated for treating patients with primary chronic Achilles tendon rupture who had a functional impairment, weakness, and palpable gap. Reconstruction of the Achilles tendon using the hamstring tendon was employed for the rupture with a gap length of at least 50 mm on magnetic resonance imaging (MRI). Preoperative MRI was performed in a relaxed supine position with natural plantar flexion of the ankle using a 1.5- or 3.0-Tesla MR system according to each scanning protocol. The proximal-distal length between the healthy tendon stumps was measured in all patients on sagittal T2-weighted MR images. Pre- and post-operative ankle range of motion (ROM), and American Orthopedic Foot and Ankle Society (AOFAS) ankle-hindfoot score was assessed, and postoperative complications were evaluated at the last follow-up as clinical outcomes.

Operative technique

**Figure 1:**
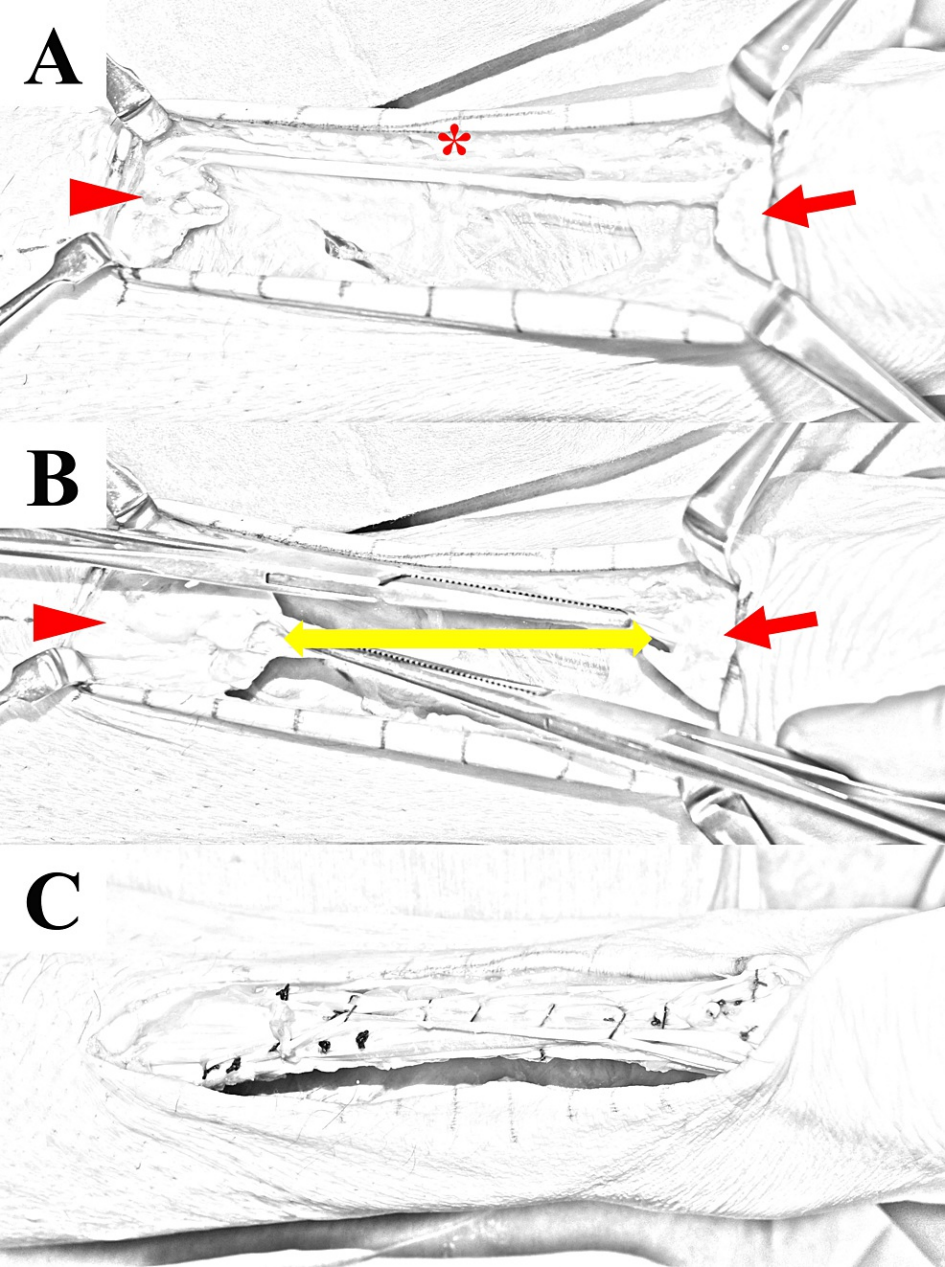
Intraoperative illustration of chronic Achilles tendon rupture. (A) Large tendon defect. Proximal (arrowhead) and distal (arrow) tendon stumps. A preserved plantaris tendon (asterisk). (B) Traction of the tendon stumps after mobilization using Kocher forceps. Tendon gap (double arrow). (C) Achilles tendon reconstruction using semitendinosus tendon. The graft is folded like square loop and sutured to fulfill the tendon defect.

All surgical procedures were performed by two orthopedic surgeons with >15 years of experience. Each surgery was performed with the patient in the prone position and under general anesthesia. A 3-cm straight incision was created over the pes anserinus, and the semitendinosus or gracilis tendon was harvested using a tendon stripper. A straight longitudinal skin incision was made along the medial border of the Achilles tendon. The proximal and distal tendon stumps were exposed (Figure 1A), and the scar tissue was resected from both tendon stumps. Adhesions were released between the proximal tendon stump and the surrounding subcutaneous tissue, and mobilization of the proximal tendon stump was confirmed under traction in the distal direction (Figure 1B). Intratendinous tendon suture was applied between both stumps using braided nylon sutures (size 1) to maintain the plantar flexion of the ankle. Using a scalpel, a slit was created at a distance of 15 mm from distal and proximal tendon stumps in the transverse direction. The hamstring tendon graft was passed into the two slits and folded like a square loop to fill the tendon defect. Then, the tendon stump and graft were sutured by a surgeon's knot in the maximal plantarflexed position using braided nylon sutures (size 0) (Figure 1C).

Postoperative management

The ankle was immobilized in the gravity-assisted equinus position for four weeks under non-weight-bearing conditions using a posterior ankle splint for a week and a below-knee cast for three weeks, respectively. Partial weight-bearing was permitted under hinged ankle-foot orthosis (AFO) with adjustable heel wedges to maintain the plantar flexed position four weeks postoperatively. ROM exercise was started as a physiotherapy program. Patients were allowed limited active ROM from gravity-assisted equinus position to plantar flex position which was defined by the angle of heel wedges. The patients were cautioned to avoid hyperdorsiflexion of the ankle. Subsequently, full weight-bearing was allowed five weeks postoperatively. Initially, four solid heel wedges were fixed on the bottom of the orthosis. One of each heel wedge was removed once a week from seven weeks to 10 weeks. The AFO was utilized to limit hyperdorsiflexion until three to four months postoperatively.

Demographic and clinical data

The demographic and clinical data of all patients are shown in Tables 1, 2. The mean duration from the traumatic event or appearance of symptoms to surgery was 3.8 (range, 2-6) months. The patients had a mean follow-up period of 40.3 (range, 23-75) months. The mean measured gap between the healthy tendon stumps was 67.5mm on preoperative sagittal T2-weighted MR images. Semitendinosus tenson graft was used for the patients with relatively large tendon gap (69mm and 79mm) compared to gracilis tendon graft (59mm and 63mm). The mean AOFAS ankle-hindfoot score improved from 67.3 to 99.5 at the final follow-up. The mean ROM was 27.0° preoperatively and 24.0° postoperatively in dorsiflexion, and 45.0° preoperatively and 42.0° postoperatively in plantar flexion. Postoperatively, a superficial incisional surgical site infection occurred in one patient with diabetes mellitus and were treated with antibiotics. No donor site morbidity was identified by measuring the hamstring muscle strength using manual muscle testing of knee flexion in all patients.

**Table 1:** Demographic and clinical data of geriatric patients Abbreviations: BMI, body mass index, N/A, not applicable.

Patients	Age	Sex	Side	BMI (kg/m^2^)	Past illness	Chief compliant	Mechanism	Chronicity (Months)	Cause of chronicity
1	78	Male	Left	25.3	N/A	Ankle pain	Long distance walking	2	Misdiagnosis
2	81	Male	Left	23.5	N/A	Difficulty in walking	Fall down	2	No visit
3	84	Male	Right	23.6	Diabetes mellitus	Pain of Achilles tendon	Fall down	5	Misdiagnosis
4	71	Male	Right	21.1	N/A	Pain of posterior calf	N/A	6	Refusal of examination
Mean	78.5			23.4				3.8	

**Table 2:** Demographic and clinical data of geriatric patients (continuation) * Postoperative AOFAS scale was obtained at the last follow-up visit (27 months after revision surgery). N/A, not applicable, AOFAS, The American Orthopaedic Foot & Ankle Society.

Patients	Location	Defect size (mm)	Graft	Follow-up periods (Months)	AOFAS scale		Complication
					Pre op.	Post op.	
1	Proximal	59	Gracilis	75	48	100	N/A
2	Midsubstance	63	Gracilis	31	85	98	N/A
3	Midsubstance	79	Semitendinosus	23	68	100	Superficial incisional surgical site infection
4	Midsubstance	69	Semitendinosus	32	68	100*	Re-rupture (Five-months follow-up)
Mean		67.5		40.3	67.3	99.5	

Three of four geriatric patients returned to daily activity at the pre-symptomatic level at four months postoperatively, and their activity level remained unchanged at the final follow-up. Re-rupture was detected at the five-month follow-up visit in another patient.

Illustrative case no. 4

A 71-year-old man started to feel pain of right posterior calf without particular cause six months earlier and was referred to our clinic. Chronic Achilles tendon rupture was detected, and he underwent reconstruction of Achilles tendon using a semitendinosus tendon graft. Ankle ROM exercise was started after a below-knee cast removal four weeks postoperatively, and partial weight bearing was permitted under AFO. Re-rupture was detected at the five-month follow-up visit, and an inquiry revealed non-use of the AFO postoperatively. As a revision surgery, the rupture site was repaired by suturing, and the Achilles tendon complex was augmented using a knotless suture anchor and suture tape (Achilles Midsubstance SpeedBridge, Arthrex, Naples, FL, USA). Although the suture site of the proximal tendon stump and graft was disrupted in this case, the hamstring tendon graft remained normal. Rehabilitation was performed according to the protocol described above. The postoperative course was good without recurrence 27 months after the revision surgery.

## Discussion

This study revealed that Achilles tendon reconstruction using hamstring tendons has the potential to improve the clinical status of older patients with large defects and the mean delay in surgery of 3.8 months in chronic Achilles tendon rupture. Nonsurgical treatment options might be reserved for patients with low functional demands or those with significant medical contraindications for surgical treatment [8]. Non-surgical management, the Swansea Morriston Achilles Rupture Treatment (SMART) protocol, yielded good midterm clinical outcomes for chronic Achilles rupture with a mean tendon gap size of 10.6 mm [13]. However, less improvement was identified in patients with a delay in treatment of >12 weeks. Thus, the SMART protocol could be an effective option even in younger and active patients, especially if the delay in treatment is <12 weeks [13].

Surgical reconstruction is appropriate management in most patients with chronic Achilles tendon rupture who have function loss, weakness, and a large gap [8]. Several reports have described good clinical outcomes of the flexor hallucis longus (FHL) tendon transfer for chronic Achilles tendon rupture using the open technique [14] or endoscopic-assisted technique [15] in patients aged 56 to 84 years. In the midterm, FHL transfer also improved the clinical status in older patients with an average gap of 4 cm between the tendon stumps [16]. However, a large tendon gap is generally clarified after debridement of the scar tissue in the chronic status. FHL transfer alone was insufficient for the reconstruction of defects > 5 cm, and combined V-Y advancement or turndown flap was required [17].

Here, the Achilles tendon ruptures transited to chronicity because the ruptures occurred with minor trauma or non-traumatically, suggesting fragility in the original Achilles tendon of older patients. Less severe degeneration is expected in the harvested graft compared to the local tissue, including the tendon and aponeurosis around the stumps, especially in older patients. Because higher degeneration was identified histologically in the ruptured Achilles tendon than in the healthy tendon [18]. Additionally, the autologous tendon graft has the potential benefit in terms of intraoperative tendon adjustment, biological processes, and better elasticity compared to the gastrocnemius aponeurosis flap [19]. Based on these knowledge, we employed the autologous hamstring tendon graft for chronic Achilles tendon rupture with a large gap size in older patients.

A superficial incisional surgical site infection was observed in one patient with diabetes mellitus as a minor complication. Re-rupture was detected in another patient at the five-month follow-up visit; however, the postoperative rehabilitation program had not been followed since the below-knee cast was removed. The patient removed the AFO without permission and started walking and performed excessive ankle dorsiflexion. The maximal strain of the Achilles tendon declines with aging, and significantly lower values were noted in participants aged approximately 70 years [20]. Thus, non-accelerated rehabilitation was applied postoperatively to older patients with chronic Achilles tendon rupture using the AFO with adjustable heel wedges to maintain the plantar flexed position during gait. Additionally, postoperative management may depend on cognitive impairment in older patients due to problems with memory, behavioral and psychological symptoms. Therefore, the postoperative rehabilitation program should be explained to the patients and their family members to allow for adequate understanding and prevention of re-rupture.

The major limitations of this study are the small number of male patients with different rupture sites such as proximal and mid substance, and the lack of a control group. We focused on older patients who accounted for a part of the patient population with chronic Achilles tendon rupture with a large tendon gap. Another technique was employed for chronic Achilles tendon rupture with a small or moderate tendon gap size. Hence, this single-center study did not address large numbers of older patients who underwent Achilles reconstruction using a hamstring tendon. Future prospective, multicenter clinical trials are warranted to investigate the midterm and long-term clinical outcomes of older male and female patients with chronic Achilles tendon rupture. Second, only the AOFAS ankle-hindfoot score, ankle ROM, and postoperative complications were assessed as clinical outcomes. Quantitative functional analysis should be evaluated in future studies, such as isometric muscle strength in plantar flexion and electromyography or tensiomyography in gastrocnemius muscles.

## Conclusions

Our results suggest that Achilles tendon reconstruction using a hamstring tendon is a viable option for treating chronic rupture of the Achilles tendon with a large tendon defect, even in older patients. Sufficient strength is expected for hamstring tendon autografting compared to the aponeurosis and tendon stumps in the chronic status, especially in older patients. To improve clinical outcomes, better understanding should be provided to family members as well as older patients regarding the postoperative rehabilitation program.
